# An Evaluation of Four Radiological Methods for Dental Age Estimation in the Montenegrin Population

**DOI:** 10.3390/diagnostics15141769

**Published:** 2025-07-13

**Authors:** Tina Pajevic, Nemanja Marinkovic, Ivan Arsic, Jovan Markovic, Petar Milovanovic, Zorana Stamenkovic, Nenad Nedeljkovic

**Affiliations:** 1 Clinic for Orthodontics, School of Dental Medicine, University of Belgrade, 11000 Belgrade, Serbia; tina.pajevic@stomf.bg.ac.rs (T.P.); ivan.arsic@stomf.bg.ac.rs (I.A.); jovan.markovic@stomf.bg.ac.rs (J.M.); zorana.stamenkovic@stomf.bg.ac.rs (Z.S.); nenad.nedeljkovic@stomf.bg.ac.rs (N.N.); 2 Laboratory for Anthropology, Institute of Anatomy, School of Medicine, University of Belgrade, 11000 Belgrade, Serbia; pmildus@gmail.com

**Keywords:** age estimation, dental age, Demirjian, Willems, BAF, European formula

## Abstract

**Objectives**: In this study, we aimed to evaluate the accuracy of four radiological methods for dental age estimation and to test which method is the most reliable in Montenegrin children. **Methods**: To determine dental age, we examined 351 panoramic radiographs of 190 female and 161 male children between the ages of 6 and 12 using the Demirjian method, Willems method, the European formula, and the BAF. The estimated dental age was compared with the chronological age, and the average, absolute, and standard deviations were calculated. **Results**: When comparing dental age to chronological age in females, overestimation occurred when using the Demirjian and Willems methods, whereas the European formula and the BAF resulted in underestimations. Only the European formula underestimated dental age in males, while the other three methods caused overestimations in comparison to chronological age. The European formula and the BAF revealed that, when all age groups were included, approximately 57.1–61% of patients deviated from their chronological age by less than six months. When using Demirjian’s method, nearly 30–35% of patients had dental ages that were either overestimated or underestimated by more than a year. **Conclusions**: The most accurate method for estimating the dental age of children under 13 in the Montenegrin population is the European formula. The Demirjian method had the lowest accuracy, whereas that in the BAF and the Willems method was similar.

## 1. Introduction

From an orthodontic perspective, dental age may provide valuable insights into the normal development of occlusions and malocclusions. During the period of mixed dentition, dental age estimation is an essential procedure in malocclusion diagnosis. Deviations in dental age from other developmental ages can help in providing treatment in the case of delayed dentition or can be another burden in the case of acceleration in dental development. In addition to use in daily orthodontic clinical practices, the estimation of dental age is an important aspect of pediatric dentistry and various scientific disciplines, including forensic dentistry, criminology (identifying undocumented individuals, for instance, in major accidents), archeology (determining the age of a person whose skeletal remains were discovered at a specific archeological location), anthropology (examining skeletal remains), and legal matters [1,2].

There are many methods of dental age estimation described in the literature. The most-used techniques analyze panoramic patient radiographs to score crown and root mineralization and the development of permanent teeth in the mandibular left quadrant, as described in the Demirjian method. This method uses eight different stages for single- and multirooted teeth based on crown and root formation. The next step is to convert the stage to an exact numerical score, which is sex-specific, and to sum the scores for the lower left quadrant to calculate dental age. Some studies have reported that this method could overestimate the exact age of patients [2,3,4,5,6]. Willems also analyzed the degree of crown and root mineralization in the first seven permanent teeth in the lower left quadrant on a panoramic radiograph. Each tooth was assigned one of eight stages of mineralization (labeled A–H). Willems’ method improved Demirjian’s original method, using the Demirjian’s score that inserted into the table that directly calculated the dental age [7]. This improvement contributed to the greater accuracy of chronological age estimation in different populations [8].

Cameriere’s method measures the internal width of open apices of the permanent teeth and tooth length. Investigations have reported the accuracy and applicability of this method, described as the European formula, in various populations [9,10,11]. On the other hand, the Kvaal method uses an intraoral radiograph of single-rooted teeth, where measurements of the tooth, pulp, and root length, as well as both root and pulp width, are made [12].

The Belgrade age formula (BAF) for dental age estimation was described a few years ago. This technique measures the total tooth length and apex width (between the inner left and right edges of the open apex) of the lower left canine and second molar with incomplete root formation [13]. Additionally, it is necessary to determine the number of permanent teeth with a closed apex in the lower left quadrant on a panoramic radiograph. All these collected parameters must be entered into sex-specific formulae for determining dental age. This technique was tested on Serbian, Italian, and Montenegrin populations, suggesting its accuracy in these populations [13,14]. Most studies compare two quantitative or qualitative methods and test their accuracy. For the Montenegrin population, no data have been published that incorporate method comparisons nor deviation between estimated dental age and chronological age. Therefore, the aim of this study was to evaluate the accuracy of four radiological methods for dental age estimation and to test which method is the most reliable in Montenegrin children.

## 2. Materials and Methods

For this retrospective cross-sectional, non-interventional study, we used 351 panoramic radiographs of children (190 girls and 161 boys, Table 1) aged 6 to 12.99 years old that were chosen randomly from the database at the Clinical Center of Montenegro in Podgorica.

Only the panoramic radiographs of patients whose Montenegrin origin was established based on their last name were examined, as the population of Montenegro also includes citizens of nearby nations, like Serbia, Croatia, and Albania. A consent document had been signed by the parent or legal guardian stating that panoramic radiography from the patient’s dental record could only be used for scientific uses and that the child’s identity would never be revealed. The children had to be healthy and without any alveolar bone pathology, prior orthodontic treatment, or systemic or developmental abnormalities that might affect the growth and development of the craniofacial complex. The exclusion criteria included poor panoramic radiography quality, dental agenesis, hyperdontia, and the presence of impacted or rotated mandibular teeth.

After checking the previously set inclusion criteria for this study, a colleague from the Clinical Center of Montenegro, who did not evaluate the dental age, recorded the patient’s sex, date of birth, date of panoramic radiography record, medical history, and identification number. The selected panoramic radiographs were sent to authors via email for further examination. All panoramic radiographs were converted to “jpeg” files, and the Image J version 1.53 program (public domain open-source software, National Institute of Mental Health Bethesda, MA, USA) was used for all necessary measurements.

All measurements and analyses were performed in the lower left quadrant. If a tooth from the lower left quadrant was missing or inapplicable, the lower right quadrant was utilized as the default.

According to the chart Demirjian proposed in his original paper, each tooth is given a mineralization stage [15], upon which each permanent tooth in the lower left quadrant is given a developmental score. Together, the developmental ratings generate a total score that is translated into dental age. In Willems’ method, each tooth receives a score from a table based on its developmental stage, which is directly stated in years [7]. The total of these scores is used to assess dental age. As described in the original publication for the European formula, we examined the morphological features of each of the seven teeth that were analyzed in order to estimate dental age [16]. Using the Image J program, we measured the width of the open apex and the height of the permanent tooth in the lower left quadrant. We determined the subjects’ dental ages by applying the measured parameters to sex-specific formulae. Estimating dental age using the BAF required the following parameters: sex, the number of fully developed teeth, apex width (the space between the mesial and distal inner side of the open apex), and tooth length in the permanent canine and second molar [13].

The estimated difference between dental and chronological age has been defined as either overestimated or underestimated based on whether the result was positive or negative.

Statistical analysis was conducted using the IBM SPSS Statistics for Windows, Version 20.0 (IBM Corp., Armonk, NY, USA). The difference between dental and chronological age was analyzed using a one-sample T-test to determine whether the deviation in the estimated dental age from the chronological age was significantly different to 0. Any *p*-value below 0.05 was considered significant. A month after the first measurements, two researchers (T.P.; N.M.) repeated their measurements on 50 randomly selected panoramic radiographs. To assess the inter- and intra-observer agreement, we used the intraclass correlation coefficient, while the differences between the estimated age and chronological age have been illustrated with a modified Bland–Altman diagram [17]. We assessed the percentage of individuals whose estimated ages deviated between 0.5 and 1 year from their actual chronological age. Post-hoc power analysis for the one-sample T-test was performed in G*Power 3.1.9.7, with an alpha level of 0.05 and an effect size of 0.02 [18].

## 3. Results

Post=hoc power analysis revealed 86% power in the female subsample and 81% power in the male subsample. The intra-observer agreement was 0.994, 0.996, 0.997, and 0.993 for Demirjian’s method, Willems’ method, the European formula, and the BAF, respectively, while the inter-observer agreement was 0.990, 0.988, 0.994, and 0.990, respectively. According to the one-sample T-test, there was a significant deviation in estimated dental age from chronological age for both sexes; the exception to this arose when the European formula and Willems’ method were used to estimate the dental age in males and females, respectively. Demirjian’s and Willems’ methods overestimated dental age in comparison to chronological age in females, whereas the European formula and the BAF underestimated it. Only the European formula underestimated dental age in males, whereas the other three methods overestimated dental age in comparison with chronological age. Table 2 illustrates the mean values of the deviation in the estimated dental age from the chronological age, standard deviation, and *p* value.

The mean and absolute values of the difference between the estimated dental age and the chronological age for each age group in both females and males are presented in Table 3 and Table 4. According to the absolute values in both males and females, there was a tendency for increased overestimation in the 7–12 age category for Demirjian’s method (Table 3 and Table 4). This pattern could not be observed when considering the absolute values of difference between the estimated dental and chronological age for the other three methods.

The percentage of patients with estimated dental age that deviate from chronological age by ±6 to 12 months using Demirjian’s and Willems’ methods, the European formula, and the BAF is shown in Table 5. When all age groups were pooled, the European formula and the BAF showed that between 57.1 and 61% of patients differ from their chronological age by up to 6 months. In Demirjian’s method, almost 30–35% of the patients had their dental age underestimated or overestimated by more than 1 year.

**Figure 1:**
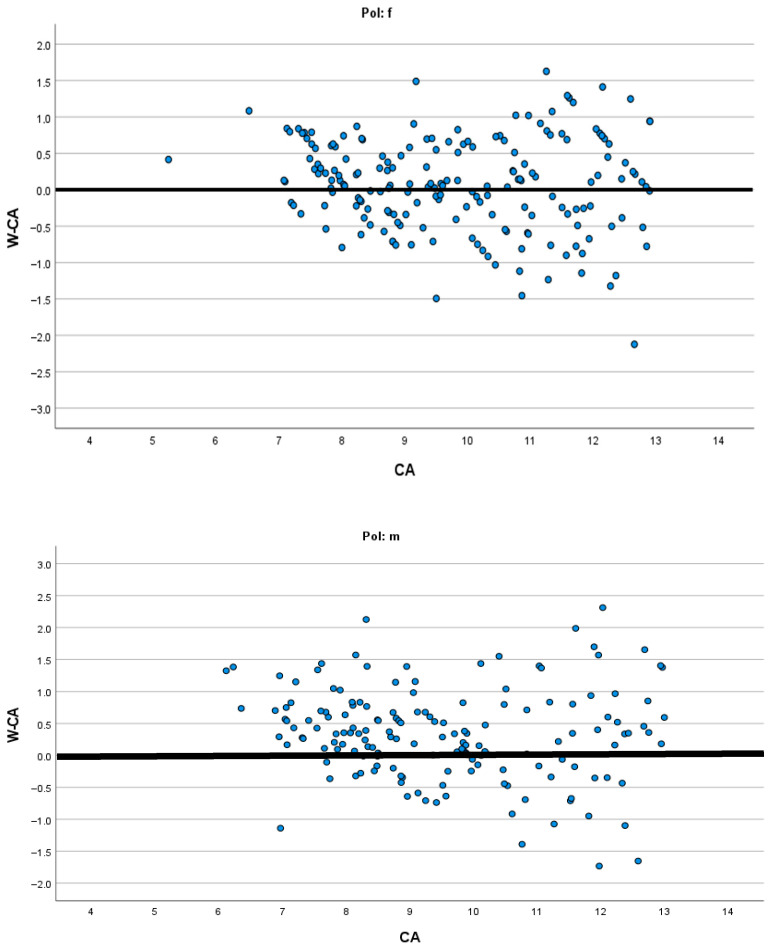
Modified Bland–Altman diagrams showing how estimated age differed from chronological age for both females and males across the whole age range when Willems’s method was applied.

**Figure 2:**
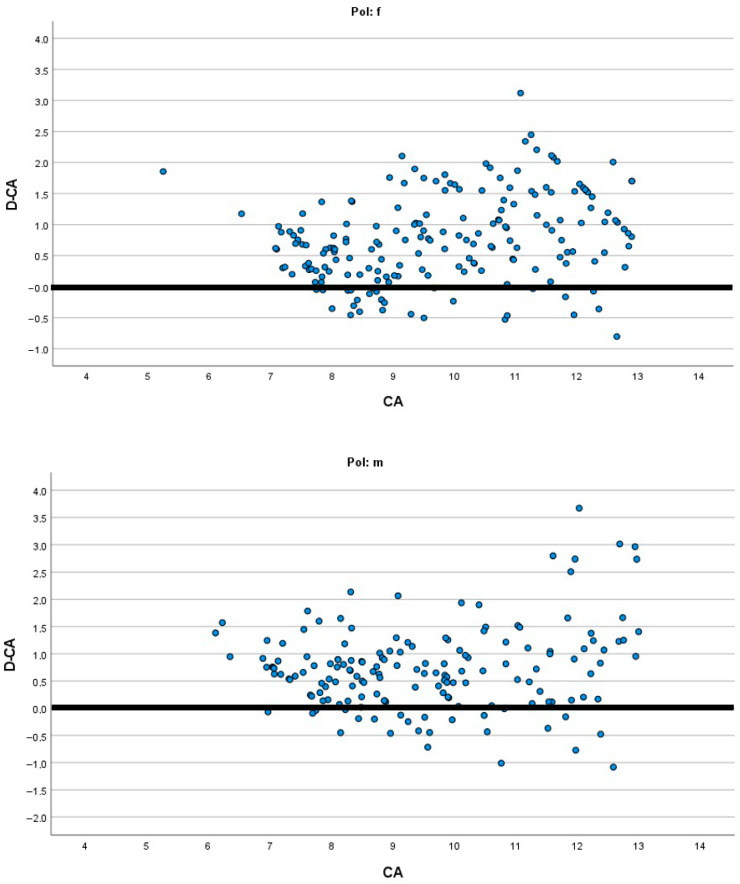
Modified Bland–Altman diagrams showing how the estimated age differed from chronological age for both females and males across the whole age range when Demirjian’s method was applied.

**Figure 3:**
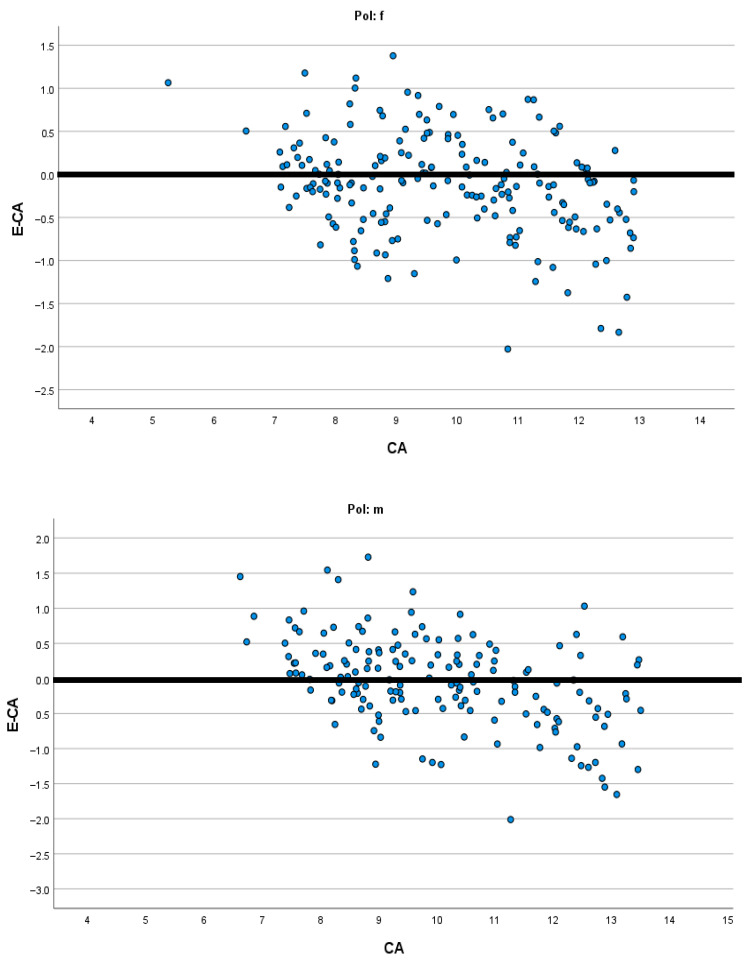
Modified Bland–Altman diagrams showing how the estimated age differed from chronological age for both females and males across the whole age range when the European formula was applied.

**Figure 4:**
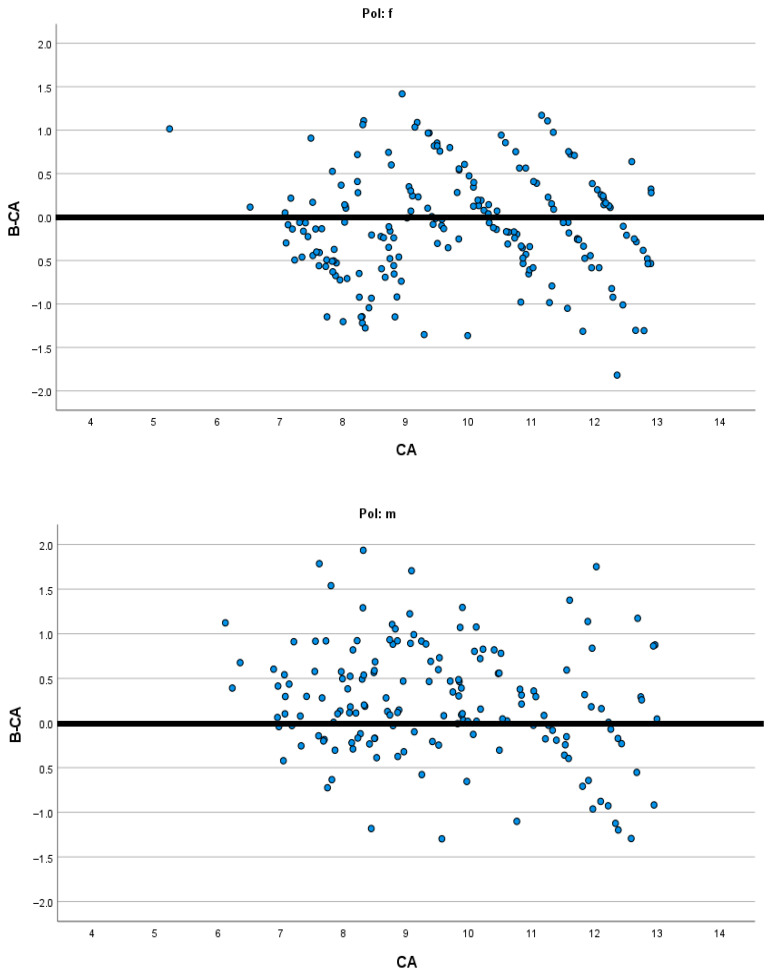
Modified Bland–Altman diagrams showing how the estimated age differed from chronological age for both females and males across the whole age range when the BAF was applied.

Figure 1, Figure 2, Figure 3 and Figure 4 present the modified Bland–Altman plots for all methods in females and males that demonstrate the distribution of the determined difference between estimated dental and chronological age across all age groups for the entire study sample.

## 4. Discussion

Dental age analysis might be a crucial diagnostic technique for determining biological maturity [19,20]. A biological age assessment is significant in orthodontic clinical practice to determine the ideal time to begin orthodontic treatment, the best type of appliance to use, and which type of treatment to apply [19]. Furthermore, other medical specialties, including pediatrics, endocrinology, and forensic medicine, could benefit from the use of dental maturity assessment. From a forensic perspective, age determination in living or deceased individuals can be utilized in a variety of circumstances, such as identifying victims of mass disasters, applying for asylum, compensating for a lack of personal documentation, adopting a child, or in cases of sexual assault, abuse, etc. [21,22,23]. Given everything mentioned above, the dental age estimation method used should be accurate and reliable. Consequently, studies testing various approaches to dental age estimation in various populations and on a sizable study sample are essential.

To the best of our knowledge, there is no published study in which four different methods for assessing dental age in the Montenegrin population have been tested and compared. This study examined the accuracy and reliability of two qualitative (Demirjian’s method and Willems’ method) and two quantitative (the European formula and the BAF) radiological approaches for dental age assessment in the Montenegrin population.

This study’s findings demonstrated that when any of the radiological techniques we employed to the Montenegrin study sample, including Demirjian’s method, Willems’ method, the European formula, or the BAF, the difference between the estimated dental age and chronological age was statistically significant. Only when Willems’ method was used on female participants, or when the European formula was used to estimate dental age in male participants, were these results not observed. Some authors stated that in forensics and anthropology, the acceptable range between estimated and chronological age is ± 1 year, while the clinically acceptable range of error in age estimation is 0.5 years [24]. Because the average deviation was less than 0.5 years, Table 2 suggests that Willems’ method, the European formula, and the BAF might be used to estimate dental age in Montenegrin children from a clinical standpoint. This suggests that, while the estimated dental age and chronological age differ statistically significantly, the discrepancy is not clinically relevant. All four methods showed that the average deviation of the estimated dental from chronological age was less than 1 year, which would mean that, from an anthropological and forensic perspective, all four radiological methods could be applied for age estimation.

According to a systematic review that examined the accuracy of Demirjian’s and Willems’ methods for estimating dental age in various populations, the former overestimated dental age compared to chronological age by an average of 0.62 years in males and 0.74 years in females, which is in line with our findings (0.77 in females and 0.72 in males) [8]. On average, Willems’ method overestimated age in males and females by 0.26 and 0.29 years, respectively, in the same study [8]. In accordance with our findings, a study by Badek et al. found that this method performed better in terms of accuracy than Demirjian’s method [25], in which the average difference between dental and chronological age was 0.80 and 0.84 years for boys and girls, respectively, and 0.41 and 0.22 years, respectively, when Willems’ method was applied [25]. Studies estimating the dental age of Turkish, Chinese, and German children using the European formula have shown that the European formula underestimated dental age in relation to chronological age, which was further confirmed in this study [26,27,28]. However, in relation to the previously mentioned studies, in the Montenegrin sample, the underestimation of dental age was slightly lower compared to other populations. Zelić et al. found that the average difference between chronological age and dental age estimated by the BAF in the Serbian validation sample was −0.149 for girls and 0.188 for boys, which is similar to the results of this study (−0.1 for girls and 0.24 for boys) [13].

If we consider the absolute deviations in the estimated dental age from the chronological age in Table 3 and Table 4, we can see that this deviation increases in older categories of participants, regardless of the method used. This may be explained by the decrease in the proportion of teeth with incomplete root mineralization, which lowers the number of factors examined in the dental age estimation process.

In a Croatian sample, when Cameriere’s European formula was applied, the percentage of boys with dental variation of ±6 and ±12 months from their chronological age was 61.5% and 87.2%, respectively [29]. These percentages are similar to the percentages for boys when the same method was applied to the Montenegrin sample (Table 5). However, for females in the Montenegrin sample, the European formula was more accurate than for the Croatian sample [29]. Demirjian’s and Willems’ methods also showed a higher percentage of subjects (both girls and boys) in whom a deviation in dental and chronological age between ±6 months and ±12 months was found. This was particularly apparent when Demirjian’s method was employed. The overall percentage of girls whose estimated dental age deviated by ±6 months was 22.6% in the Croatian sample and 35.8% in the Montenegrin sample [29]. The fact that there were many more female participants in the Croatian sample than in the Montenegrin sample could be one explanation for this. However, in both the Croatian and Montenegrin samples, the European formula revealed the highest percentage of participants whose estimated age did not fluctuate by more than ±6 months and ±12 months, while Demirjian’s method revealed the lowest percentage. This might suggest that when measuring dental age in Montenegrin children under the age of 12, the European formula could have the highest accuracy and reliability.

In a study by Dervišević et al., the percentage of participants in whom the difference between the estimated dental and chronological age was no more than 6 months using the European formula was 47.8 for boys and 44.5 for girls, which was lower than that in our Montenegrin study sample (60.9% and 61% for boys and girls, Table 5) [30]. When comparing the Montenegrin sample to Bosnian and Herzegovina participants, the BAF performed better when we looked at age deviations of both ± 6 and ± 12 months.

The modified Bland–Altman diagrams showed the dispersion of the differences between estimated dental and chronological age for the investigated methods. There is a markedly higher frequency of overestimation using the Demirjian method in both males and females (Figure 2). This is in agreement with the findings of Hostiuc et al., who reported overestimation resulting from Demirjian’s method in children aged from 3–4 to 14–15 years [31]. On the contrary, the authors of the systematic review found that, above 15 years, until the age of 19, there is a tendency for underestimation using the Demirjian method. In the other three methods, there is almost an equal distribution of overestimation and underestimation (Figure 1, Figure 3 and Figure 4), while in all methods there is higher data dispersion in males compared to females; this means that all methods are more accurate in females. Dispersion of age differences varies among the age categories. All four methods appear more precise at the lower ages, from 7 to 10 years, while in older groups there is a higher dispersion of differences between estimated dental age and chronological age. In comparison to Demirjian’s and Cameriere’s methods, Darıcı et al. reported the overestimation of Demirjian’s method in all child age groups, except for 8-year-old girls, while Cameriere’s method underestimated dental age for the whole sample, except for 6-year-old children [32]. In our study, the European formula underestimated dental age in 10–12-year olds, but in children aged 7–9, it overestimated dental age.

The limitations of this research could include its small size sample, the unequal distribution of male and female participants in specific age groups (10, 11, and 12 years), and the limited number of participants in the 6–6.99 age group. That being said, it should be noted that there are only over 600,000 people living in Montenegro, making it more challenging to obtain panoramic radiographs of younger children. Due to the small number of participants who were younger than seven years old, the results were not calculated and presented in Table 3 and Table 4, unlike the other results. Future research could examine new models proposed by various authors for dental age assessment, as well as the potential applications of artificial intelligence in this field [33,34].

## 5. Conclusions

We conclude from this study’s findings that the European formula is the most reliable method of determining the dental age of children under 13 years old in the Montenegrin population. The BAF and Willems’ method demonstrated comparable accuracy, while the accuracy of Demirjian’s method was the lowest.

## Figures and Tables

**Table 1:** Distribution of male and female individuals in all age categories in the final study sample.

Age Category	Male	Female
6.00–6.99	7	2
7.00–7.99	27	31
8.00–8.99	39	38
9.00–9.99	30	31
10.00–10.99	18	34
11.00–11.99	21	28
12.00–12.99	19	26

**Table 2:** Mean difference between chronological and estimated dental age according to Willems’ method, Demirjian’s method, the European formula, and the BAF.

Estimated DA ^1^—CA ^2^	Mean	Std. Deviation ^3^	*p* Value
females			
W ^4^—CA	0.08	0.64	0.07
D ^5^—CA	0.77	0.70	0.00 *
E ^6^—CA	−0.12	0.58	0.00 *
B ^7^—CA	−0.10	0.61	0.02 *
males			
W—CA	0.33	0.70	0.00 *
D—CA	0.72	0.77	0.00 *
E—CA	−0.04	0.64	0.39
B—CA	0.24	0.63	0.00 *

^1^ DA—dental age; ^2^ CA—chronological age; ^3^ Std. deviation—standard deviation; ^4^ W—dental age estimated by the Willems’ method; ^5^ D—dental age estimated by Demirjian’s method; ^6^ E—dental age estimated with the European formula; ^7^ B—dental age estimated with the BAF; * *p* value < 0.05.

**Table 3:** The mean and absolute values of the difference between estimated dental and chronological age for each age category in females.

Age Category	CA ^1^ Mean ^6^ ± SD ^7^	W ^2^—CA Mean ± SD	W—CA ABS ^8^ ± SD	D ^3^—CA Mean ± SD	D—CA ABS ± SD	E ^4^—CA Mean ± SD	E—CA ABS ± SD	BAF ^5^—CA Mean ± SD	BAF—CA ABS ± SD
7–7.99	7.56 ± 0.31	0.32 ± 0.38	0.41 ± 0.26	0.51 ± 0.35	0.52 ± 0.34	0.04 ± 0.39	0.29 ± 0.26	−0.26 ± 0.40	0.41 ± 0.26
8–8.99	8.49 ± 0.30	−0.04 ± 0.45	0.37 ± 0.25	0.32 ± 0.55	0.48 ± 0.40	−0.16 ± 0.64	0.53 ± 0.38	−0.30 ± 0.71	0.65 ± 0.40
9–9.99	9.46 ± 0.28	0.11 ± 0.58	0.43 ± 0.40	0.85 ± 0.70	0.93 ± 0.59	0.12 ± 0.54	0.44 ± 0.33	0.24 ± 0.61	0.50 ± 0.42
10–10.99	10.56 ± 0.31	−0.10 ± 0.65	0.53 ± 0.37	0.86 ± 0.62	0.94 ± 0.53	−0.16 ± 0.52	0.42 ± 0.37	−0.01 ± 0.45	0.37 ± 0.26
11–11.99	11.52 ± 0.28	0.08 ± 0.82	0.70 ± 0.43	1.18 ± 0.90	1.22 ± 0.83	−0.19 ± 0.61	0.52 ± 0.36	−0.02 ± 0.65	0.53 ± 0.37
12–12.99	12.47 ± 0.30	0.15 ± 0.84	0.68 ± 0.50	1.01 ± 0.69	1.10 ± 0.51	−0.50 ± 0.56	0.54 ± 0.53	−0.30 ± 0.61	0.51 ± 0.44

^1^ CA—chronological age; ^2^ W—dental age estimated by Willems’ method; ^3^ D—dental age estimated by Demirjian’s method; ^4^ E—dental age estimated with the European formula; ^5^ BAF—dental age estimated with the BAF; ^6^ Mean—mean value; ^7^ SD—standard deviation; ^8^ ABS—absolute value.

**Table 4:** The mean and absolute values of difference between estimated dental and chronological age for each age category in males.

Age Category	CA ^1^ Mean ^6^ ± SD ^7^	W ^2^—CA Mean ± SD	W—CA ABS ^8^ ± SD	D ^3^—CA Mean ± SD	D—CA ABS ± SD	E ^4^—CA Mean ± SD	E—CA ABS ± SD	BAF ^5^—CA Mean ± SD	BAF—CA ABS ± SD
7–7.99	7.55 ± 0.38	0.53 ± 0.43	0.56 ± 0.38	0.65 ± 0.46	0.66 ± 0.44	0.28 ± 0.50	0.41 ± 0.40	0.26 ± 0.60	0.48 ± 0.44
8–8.99	8.48 ± 0.28	0.37 ± 0.59	0.52 ± 0.47	0.57 ± 0.55	0.64 ± 0.46	0.02 ± 0.54	0.41 ± 0.34	0.31 ± 0.58	0.49 ± 0.42
9–9.99	9.57 ± 0.31	0.15 ± 0.49	0.39 ± 0.31	0.52 ± 0.62	0.67 ± 0.44	0.03 ± 0.63	0.50 ± 0.37	0.37 ± 0.63	0.58 ± 0.45
10–10.99	10.42 ± 0.27	0.11 ± 0.78	0.59 ± 0.51	0.67 ± 0.80	0.85 ± 0.60	−0.13 ± 0.61	0.41 ± 0.47	0.32 ± 0.53	0.49 ± 0.36
11–11.99	11.53 ± 0.32	0.25 ± 1.01	0.85 ± 0.59	0.85 ± 0.99	0.98 ± 0.86	−0.38 ± 0.52	0.54 ± 0.35	0.06 ± 0.58	0.43 ± 0.38
12–12.99	12.51 ± 0.32	0.45 ± 0.93	0.82 ± 0.60	1.26 ± 1.19	1.42 ± 0.98	−0.56 ± 0.74	0.78 ± 0.49	−0.10 ± 0.86	0.67 ± 0.51

^1^ CA—chronological age; ^2^ W—dental age estimated by Willems’ method; ^3^ D—dental age estimated by Demirjian’s method; ^4^ E—dental age estimated with the European formula; ^5^ BAF—dental age estimated with the BAF; ^6^ Mean—mean value; ^7^ SD—standard deviation; ^8^ ABS—absolute value.

**Table 5:** The percentage of individuals with an estimated dental age that differed from chronological age between ±6- and 12 months using Willems’s method, Demirjian’s method, the European formula, and the BAF.

Dental Age Estimation Methods	Sex	Underestimation/Overestimation of Dental Age	
		±6 Months *	±12 Months **
Willems	Females	52.1%	89.5%
	Males	50.3%	80.1%
Demirjian	Females	35.8%	65.3%
	Males	35.4%	70.2%
European formula	Females	61.0%	91.1%
	Males	60.9%	88.2%
BAF	Females	57.9%	87.4%
	Males	57.1%	86.3%

* Percentage of patients in whom the difference between estimated dental and chronological age was less than 6 months. ** Percentage of patients in whom the difference between estimated dental and chronological age was less than 12 months.

## Data Availability

The dataset is available on request from the authors.

## References

[1] Alrashidi M., Al-Moshiqah A., Kolarkodi S.H., Alotaiby F. (2023). A Comparative Study of the Assessment of the Accuracy of Willems’ and Demirjian’s Methods in Dental Age Estimation of the Saudi Arabian Population. Cureus.

[2] Khorate M.M., Dinkar A.D., Ahmed J. (2014). Accuracy of age estimation methods from orthopantomograph in forensic odontology: A comparative study. Forensic Sci. Int..

[3] Flood S.J., Franklin D., Turlach B.A., McGeachie J. (2013). A comparison of Demirjian’s four dental development methods for forensic age estimation in South Australian sub-adults. J. Forensic Leg. Med..

[4] Feijóo G., Barbería E., De Nova J., Prieto J.L. (2012). Dental age estimation in Spanish children. Forensic Sci. Int..

[5] Celik S., Zeren C., Çelikel A., Yengil E., Altan A. (2014). Applicability of the Demirjian method for dental assessment of southern Turkish children. J. Forensic Leg. Med..

[6] Altan H., Altan A., Sozer O.A. (2017). Dental age estimation in southern Turkish children: Comparison of Demirjian and Willems methods. Inn. J. Pediatr..

[7] Willems G., Van Olmen A., Spiessens B., Carels C. (2001). Dental age estimation in Belgian children: Demirjian’s technique revisited. J. Forensic Sci..

[8] Esan T.A., Yengopal V., Schepartz L.A. (2017). The Demirjian versus the Willems method for dental age estimation in different populations: A meta-analysis of published studies. PLoS ONE.

[9] Melo M., Ata-Ali F., Ata-Ali J., Martinez Gonzalez J.M., Cobo T. (2022). Demirjian and Cameriere methods for age estimation in a Spanish sample of 1386 living subjects. Sci. Rep..

[10] Hostiuc S., Diaconescu I., Rusu M.C., Negoi I. (2021). Age Estimation Using the Cameriere Methods of Open Apices: A Meta-Analysis. Healthcare.

[11] Marinkovic N., Milovanovic P., Djuric M., Nedeljkovic N., Zelic K. (2018). Dental maturity assessment in Serbian population: A comparison of Cameriere’s European formula and Willems’ method. Forensic Sci. Int..

[12] Kvaal S.I., Kolltveit K.M., Thomsen I.O., Solheim T. (1995). Age estimation of adults from dental radiographs. Forensic Sci. Int..

[13] Zelic K., Marinkovic N., Milovanovic P., Cameriere R., Djuric M., Nedeljkovic N. (2020). Age estimation in children based on open apices measurement in the Serbian population: Belgrade Age Formula (BAF). Ann. Hum. Biol..

[14] Marković J., Marinković N., Arsić I., Zelić K., Stamenković Z., Glamočanin B., Nedeljković N. (2022). The accuracy of Belgrade Age Formula method for dental age estimation in Montenegrin children aged 10–12 years. Srp. Arh. Celok. Lek..

[15] Demirjian A., Goldstein H., Tanner J.M. (1973). A new system of dental age assessment. Hum. Biol..

[16] Cameriere R., Ferrante L., Cingolani M. (2006). Age estimation in children by measurement of open apices in teeth. Int. J. Leg. Med..

[17] Krouwer J.S. (2008). Why Bland-Altman plots should use X, not (Y+X)/2 when X is a reference method. Stat. Med..

[18] Faul F., Erdfelder E., Lang A.-G., Buchner A. (2007). G*Power 3: A flexible statistical power analysis program for the social, behavioral, and biomedical sciences. Behav. Res. Methods.

[19] Liversidge H.M., Buckberry J., Marquez-Grant N. (2015). Age estimation. Ann. Hum. Biol..

[20] Milani S., Benso L. (2019). Why we can’t determine reliably the age of a subject on the basis of his maturation degree. J. Forensic Leg. Med..

[21] Cameron N. (2015). Can maturity indicators be used to estimate chronological age in children?. Ann. Hum. Biol..

[22] Levinson A. (2011). Unaccompanied Immigrant Children: A Growing Phenomenon with Few Easy Solutions [Internet]. https://www.migrationpolicy.org/article/unaccompanied-immigrant-children-growing-phenomenon-few-easy-solutions.

[23] Crawley H. (2000). When Is a Child Not a Child? Asylum, Age Disputes and the Process of Age Assessment. https://www.academia.edu/25307504/When_is_a_Child_not_a_Child_Asylum_Age_Disputes_and_the_Process_of_Age_Assessment.

[24] Flood S.J., Mitchell W.J., Oxnard C.E., Turlach B.A., McGeachie J. (2011). A comparison of Demirjian’s four dental development methods for forensic age assessment. J. Forensic Sci..

[25] Bedek I., Dumančić J., Lauc T., Marušić M., Čuković-Bagić I. (2022). Applicability of the Demirjian, Willems and Haavikko methods in Croatian children. J. Forensic Odontostomatol..

[26] Sezer B., Çarıkçıoğlu B. (2022). Accuracy of the London Atlas, Haavikko’s Method and Cameriere’s European Formula of dental age estimation in Turkish children. Leg. Med..

[27] Yang Z., Wen D., Xiao J., Liu Q., Sun S., Kureshi A., Chang Y., Zha L. (2021). Application of Cameriere’s method for dental age estimation in children in South China. Forensic Sci. Res..

[28] Halilah T., Khdairi N., Jost-Brinkmann P.G., Bartzela T. (2018). Age estimation in 5-16-year-old children by measurement of open apices: North German formula. Forensic Sci. Int..

[29] Brkić H., Galić I., Vodanović M., Dumančić J., Mehdi F., Anić Milošević S. (2022). The Cameriere, Haavikko, Demirjian, and Willems methods for the assessment of dental age in Croatian children. Int. J. Legal Med..

[30] Dervišević E., Selmanagić A., Milovanović P., Zelić-Mihajlović K. (2024). Age Determination Based on Open Apex Measurement in the Developing Dentition: Comparing the Accuracy of the Belgrade Age Formula (BAF) with the European Formula on a Bosnian Children Population. Acta Stomatol. Croat..

[31] Hostiuc S., Edison S.E., Diaconescu I., Negoi I., Isaila O.M. (2021). Accuracy of the Demirjian’s method for assessing the age in children, from 1973 to 2020. A meta-analysis. Leg. Med..

[32] Darıcı A., Ölmez M.S., Güngör H.C., Rajavaara P., Sipola A., Anttonen V., Päkkilä J. (2024). Comparison of accuracy of different dental age estimation methods in Finnish and Turkish populations. Acta Odontol. Scand..

[33] Bedek I., Dumančić J., Lauc T., Marušić M., Čuković-Bagić I. (2020). New model for dental age estimation: Willems method applied on fewer than seven mandibular teeth. Int. J. Legal Med..

[34] Vila-Blanco N., Varas-Quintana P., Tomás I., Carreira M.J. (2023). A systematic overview of dental methods for age assessment in living individuals: From traditional to artificial intelligence-based approaches. Int. J. Legal Med..

